# Social contact, practice, organization and technical knowledge: Experiences of music students in the course of the COVID-19 pandemic

**DOI:** 10.3389/fpsyg.2022.885890

**Published:** 2022-09-14

**Authors:** Claudia Spahn, Anna Immerz, Anna Maria Hipp, Manfred Nusseck

**Affiliations:** Freiburg Institute for Musicians’ Medicine, University of Music Freiburg, Medical Faculty of the Albert-Ludwigs-University Freiburg, Freiburg Center for Research and Teaching in Music, Freiburg, Germany

**Keywords:** personal experiences, social contact, organization, technical knowledge, music students, coronavirus pandemic

## Abstract

For music students, the COVID-19 pandemic has had a great impact, forcing them to adapt to certain coronavirus regulations laid down by the state. In this study, the experiences of music students in three consecutive semesters under different coronavirus-related conditions are investigated. At the end of three semesters, the lockdown semester [SS (Summer Semester) 2020: April – July], a partially opened semester [WS (Winter Semester) 2020/21: October – February] and a mostly opened semester (SS 2021), a total of 152 music students at the University of Music Freiburg were asked to fill in an online survey. A mixed-methods approach was used, with results showing that the qualitative statements of the students support the quantitative data. The results of the cross-sectional study demonstrate that self-regulated learning improved during the lockdown semester, through new time management and focused practice with regular breaks. During the partially opened semester, the use of blended learning formats led to organizational problems, such as travel time and change of locations. Furthermore, music students were challenged by the social distancing, which improved during the partially opened, and mostly opened semester. New technologies regarding digital communication formats were emerged, which have evolved over the course of the three semesters. Concerning the overall experience, students stated that the partially-opened semester was most challenging, since distances and change of locations had to be combined with quickly changing public COVID-19-regulations. These findings during different stages of the COVID-19 pandemic provide constructive starting points for future teaching.

## Introduction

Since the Summer Semester (SS) 2020, the situation at universities has been markedly affected by the COVID-19 pandemic. During that semester and the following two, certain coronavirus-related rules had to be followed (e.g., regular ventilation, wearing of facemasks, distancing, restrictions in using the university buildings) and teaching at universities changed significantly, too. For music students, the situation largely concerned the use of practice rooms and the instrumental and vocal lessons.

Recent studies show that students, in general, have developed a range of approaches to meet the different conditions of the coronavirus pandemic (Biasutti et al., 2021; Habe et al., 2021; Holm-Hadulla et al., 2021; Martinek et al., 2021; Nusseck and Spahn, 2021; Rosset et al., 2021; Schiavio et al., 2021; Tejedor et al., 2021).

One topic, that students of all disciplines had to find ways to deal with, are online courses. Universities of Music have had less experience in online teaching, compared to the long-standing tradition of face-to-face artistic instruction, which clearly requires a certain interaction between teacher and learner (de Bruin, 2021; Güsewell and Terrien, 2021; Vladova et al., 2021). Organizational aspects were found to be problematic, whereas acceptance of technical requirements (such as programs for online course) on the part of both teachers and learners was mostly high. Li et al. (2021) observed the use of online courses at a Chinese performing arts university during times of social isolation, specifically, hybrid teaching formats such as blended learning, mixed teaching methods, and flipped classrooms, in combination with outcome-based and student-oriented learning. It was shown that these formats if employed in a professional way can lead to rich and meaningful teaching and learning. Güsewell and Terrien (2021) observed the relationship, challenges and chances of one-on-one teaching in musical higher education in Switzerland and France, looking mainly at the year of 2020. They did an online survey (*N* = 56), as well as semi-structured interviews with 3 teachers. Difficulties that were mentioned were connection problems with students, poor network, unsuitable or missing tools, poor sound quality, high levels of stress and discomfort, and a very time-consuming nature of online courses. On the other side, they also found positive effects, such as an increase in the pedagogy level, as well as interesting spill-overs for their teaching. The study of Ozer and Ustun (2020) took place in the beginning of the COVID-19 pandemic in 2019/2020, focusing on students’ experiences with the lockdown semester, and especially distance education in the Music and Performing Arts at Nevsehir Hacı Bektas Veli University, Turkey. The students reported mainly difficulties with distance education, with the exception of music students, who found that they were able to self-improve in individual courses and were able to work more efficiently, while having more time alone. Rucsanda et al. (2021) observed the attitude towards online lessons in individual courses of music students at a University of Music in Romania during the lockdown semester. They found that by the compulsion of using e-learning tools, the attitude towards them changed and led to more satisfaction.

Holm-Hadulla et al. (2021) investigated the personal experiences of students at the University of Heidelberg during the COVID-19 pandemic. They found that loneliness and social isolation were the main reasons for a decrease in students’ well-being, along with a loss of motivation.

A study of 310 sports and music students was conducted by Habe et al. (2021), in which lockdown conditions were compared with those pre-coronavirus. Researchers found that sport students experienced predominantly positive effects compared to music students. At the same time, they advance the hypothesis that music students were able to deal with the lockdown and the accompanying social isolation more effectively especially since they are used to being alone when practicing.

In their qualitative study, Schiavio et al. (2021) addressed the special situation in music studies. Questioning music students at an Italian conservatory, they found that approaches to practicing and time management have improved during the COVID-19 pandemic. In particular, prioritizing short-and long-term goals, enhancing creative potential and building interactions with peers were found to be core themes. A further related issue that music students faced during the pandemic is the development of certain self-regulated learning skills (McPherson et al., 2017; Nusseck and Spahn, 2021). In this context, Nusseck and Spahn (2021) observed that students spent less time practicing during the lockdown semester, but they also developed new, more self-regulated practicing methods, which led to greater efficiency.

For music students in particular the relationship to their teachers is of especial importance, since it is mainly one-on-one teaching. The lockdown semester brought changes to this interpersonal relationship (Antonini Philippe et al., 2020) in terms of social distancing. The music students began to rethink the relative importance of this relationship, developing more self-regulated practicing behaviors, resulting in a better understanding of their own learning and practicing. Rosset et al. (2021) also investigated the impact of the coronavirus pandemic on the practice behavior of music students during the lockdown semester of summer 2020. They found that music students practiced less hours with a perceived increase in stressful thoughts and feelings.

Summarizing the studies mentioned above, it becomes clear that they either examine solely the lockdown semester, or compare different regulations and their impacts on students to a time before these conditions. Therefore, they offer insight into students’ overall experiences and practice behaviors during this specific period, but deal with a potentially unreliable retrospective recall bias. Our more differentiated study aims to investigate the personal experiences and practice behaviors of music students, under varying coronavirus-related conditions, comparing three points in time: full lockdown (SS 2020), hybrid/ partially open (WS 2020/21) and mostly open (SS 2021). During each of these time frames, different social, coronavirus-related regulations prevailed, leading to different underlying conditions at the University of Music Freiburg for both students and teaching faculty.

## Materials and methods

### Design

Conceived as cross-sectional, the study contains three surveys carried out in consecutive semesters during the coronavirus pandemic, the results of which are compared with each other. The study follows a mixed-methods approach in which quantitatively evaluable items and free text answers in a questionnaire were filled out by music students (Flick, 2019). In the analyses, quantitative and qualitative data were considered jointly.

**Figure 1:**
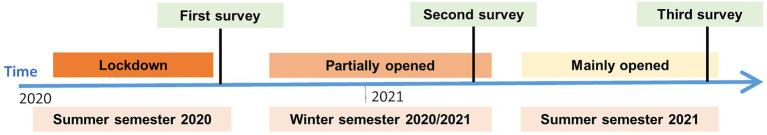
Overview of the conducted surveys on a time line.

At the University of Music Freiburg, the regulations and measures (Spahn and Richter, 2021) changed drastically between the first three semesters during the COVID-19 pandemic according to the respective stage of said regulations. We chose to collect the data at the end of each semester to better control the recall bias. The question of how students are faring under these differing coronavirus-related conditions drove the study, with three points in time compared (Figure 1): (1) July 2020: looking back on the lockdown semester (2) February 2021: looking back on partially re-opening and so-called hybrid conditions, and (3) July 2021: looking back on a mostly opened semester.

1. The first survey was carried out in July 2020, at the end of the 2020 summer semester, reviewing the lockdown semester just past. During this semester, students had nearly no courses in which their physical presence was required. It was stipulated that the courses, both theoretical and practical, be conducted in digital format. During most of the semester, the University of Music was closed. The administration and technical support staff were allowed to enter the building, with practice rooms kept open for students who were unable to practice at home. Students needed to apply for admittance to the building and had to make appointments to use a practice room. Some instrumental and vocal lessons were held in the concert halls. No gatherings at all were allowed in the building.
2. The second survey took place in February 2021, at the end of the 2020/2021 winter semester, looking back on the partially opened semester. At the University of Music Freiburg this semester was conducted in a semi-open manner. Based on the frequently updated “risk assessment of a coronavirus infection in the field of music” (last update: January 13, 2022) of the Freiburg Institute for Musicians’ Medicine, hygiene and safety measures were evaluated, which led to regulations that allowed a partial reopening of the university. Empirically verified ventilation measures plus compliance with the particular instrumental and singing-specific distancing, masking, and hand and surface cleaning rules formed the prescribed measures for teachers, students and the whole staff of the University of Music Freiburg. On this basis, lessons could be held in large rooms of the University. Especially one-on-one voice and instrumental lessons, as well as small ensembles and chamber music groups with students and their teachers, were allowed to take place. Furthermore, the students had access to the University building again, and were allowed to use practice rooms. Theoretical courses were still conducted in digital format.
3. The third survey was performed in July 2021, at the end of the 2021 summer semester, looking back on the mostly open semester. This was the first semester that allowed almost normal scenarios of practicing, lessons, rehearsals and concerts with a limited number of viewers for large ensembles, choirs and orchestras, too, again drawing on empirically verified measures of ventilation, distancing, masking, and hand and surface cleaning; the increasing rates of vaccinations also played a role in this relative loosening of rules. The University of Music Freiburg enabled vaccinations for students, teachers and staff members of the university, from early 2021 on. On the basis of a survey with voluntary responses given at the end of summer semester 2021, it was found that 92% of all university members had had their first vaccination (Spahn and Richter, 2021).

For a general comparison of the three time points of the surveys, the Oxford COVID-19 Stringency Index (Ritchie et al., 2020; Hale et al., 2021) considering the degree of governmental regulations were taken into account. The value of the index ranges between zero (no regulations) and 100. In Germany, the value was in the SS 2020 between 77 in April 2020 and 55 in July 2020. At the time of the first survey, the index was at 63. In the WS 20/21, the value ranged between 50 in October 2020 and 85 in January 2021. At the time of the second survey in February 2021, the value was 83. At the beginning of the SS 2021 in April, the index was at 75 and reduced to 68 at the third survey in July 2021.

The study was performed in online surveys using the platform SoSciSurvey. The participation in the surveys was purely voluntary and music students had to affirm their participation on the first page of the survey. The data was collected anonymously and the procedure was approved by the Ethics Committee of the Medical Center of the University of Freiburg.

### Participants

For the surveys, music students of different theory practice seminars at the University of Music Freiburg were asked in the last session at the end of the semester to participate in the survey as part of an evaluation of these courses. The seminars were regular common courses in the area of music physiology offered for all students across all study areas. Therefore, the samples of this study are representative of the full spectrum of music students at the University of Music.

A total of 152 music students took part in the three surveys at the University of Music Freiburg. 56% of the 152 students were in the Bachelor of Music cohort, 40% in the Master of Music and 4% in other study profiles cohort; 73% were female students. The mean number of semesters across the 152 students was 3.8 semesters (SD = 1.9 semesters).

The characteristics of the participants in each of the three surveys can be seen in Table 1. In the first survey in SS 2020, 68 students participated, the second survey in WS 2020/2021 contained 56 students and the third survey in SS 2021 had a smaller sample size of 28 students. The distributions of gender and study profile did not significantly differ between the surveys [Gender: *χ*^2^(2) = 0.49, n.s.; Study profile: *χ*^2^(4) = 6.0, n.s.]. There was also no significant difference in the number of semesters between the surveys [*F*(2,140) = 1.94, n.s.].

**Table 1:** Characteristics of the student samples in the surveys.

		Survey 1 SS 2020 *N* = 68	Survey 2 WS 20/21 *N* = 56	Survey 3 SS 2021 *N* = 28
Age (years) Mean (SD)		23.8 (3.6)	22.5 (2.6)	24.8 (4.5)
Gender (female)		74% (*N* = 50)	75% (*N* = 42)	68% (*N* = 19)
Number of semesters Mean (SD)		4.1 (2.0)	3.4 (1.6)	4.0 (1.9)
Study profile				
Bachelor of Music		60% (*N* = 41)	59% (*N* = 33)	39% (*N* = 11)
Master of Music		35% (*N* = 24)	39% (*N* = 22)	50% (*N* = 14)
not specified		5% (*N* = 3)	2% (*N* = 1)	11% (*N* = 3)

The instruments were distributed as follows: 23% piano, 14% vocals, 33% strings, 8% brass, 15% woodwind and 7% other instruments in the total sample (*N* = 152). The distribution of the instrumental groups did not significantly differ between the surveys [*χ*^2^(8) = 8.5, n.s.].

### Questionnaire

The questionnaire contains sociodemographic data such as age, gender, number of semesters, study profile and main instrument/voice. The questionnaire was conducted in German and the students’ statements were translated into English, after the analysis.

#### Practice time

The participants were asked to estimate in minutes the amount of time spent practicing daily over the past semester. Additionally, they were asked to compare this practice time to the usual practice time in a non-coronavirus semester on a scale from 1: much less to 5: much more.

#### Practice behavior

Participants were asked to indicate whether their practice behavior had changed in the past semester compared to the previous semester and if yes, to describe in a freely formulated text how such practice behavior had changed.

#### Rating of overall experience

Students were asked to evaluate their experience of that semester compared to semesters prior to the coronavirus pandemic. The question was “How did you experience this semester compared to your studies before the Corona pandemic?” Students who started their study during the pandemic were told to skip this question. Answers were possible on a 100-point analog scale (1: negative to 100: positive). The scale allowed that a response of 50 implies a neutral value regarding a similar experience in comparison to other semesters. Moreover, students were asked to compare their overall experience of a semester with that of the previous one. The same question and scale were used, but the reference of comparison was the last semester. In the first survey, there was only one question, since the semester fell together with the earlier semester unaffected by coronavirus.

#### Open experience responses

Students were asked to give a personal statement in an open text field, about what they enjoyed most, and what they liked less about the past semester.

### Evaluation methods and statistics

#### Quantitative data

All parametric values were reported descriptively with mean and standard deviation of the mean (SD). For the analysis of main effects between the surveys, an analysis of variance (ANOVA) was used. A post-hoc analysis was performed with the Tukey-HSD correction. Nonparametric comparisons made use of cross-tables; Pearson’s *χ*^2^ was reported. The statistical analyses were conducted with SPSS (Version 28, Armonk, NY: IBM Corp.). The level of significance was set to *p* = 0.05.

#### Qualitative data

All open-text answers of the questionnaire were analyzed with the qualitative content analysis according to Mayring (2010). First, transparency was created by describing the sample in detail. Then, a category system was developed bottom up on the basis of the music students’ answers. This served as a guideline in the course of further interpretation. Through this procedure and by working jointly in a research team, reproducibility and objectivity were guaranteed.

**Figure 2:**
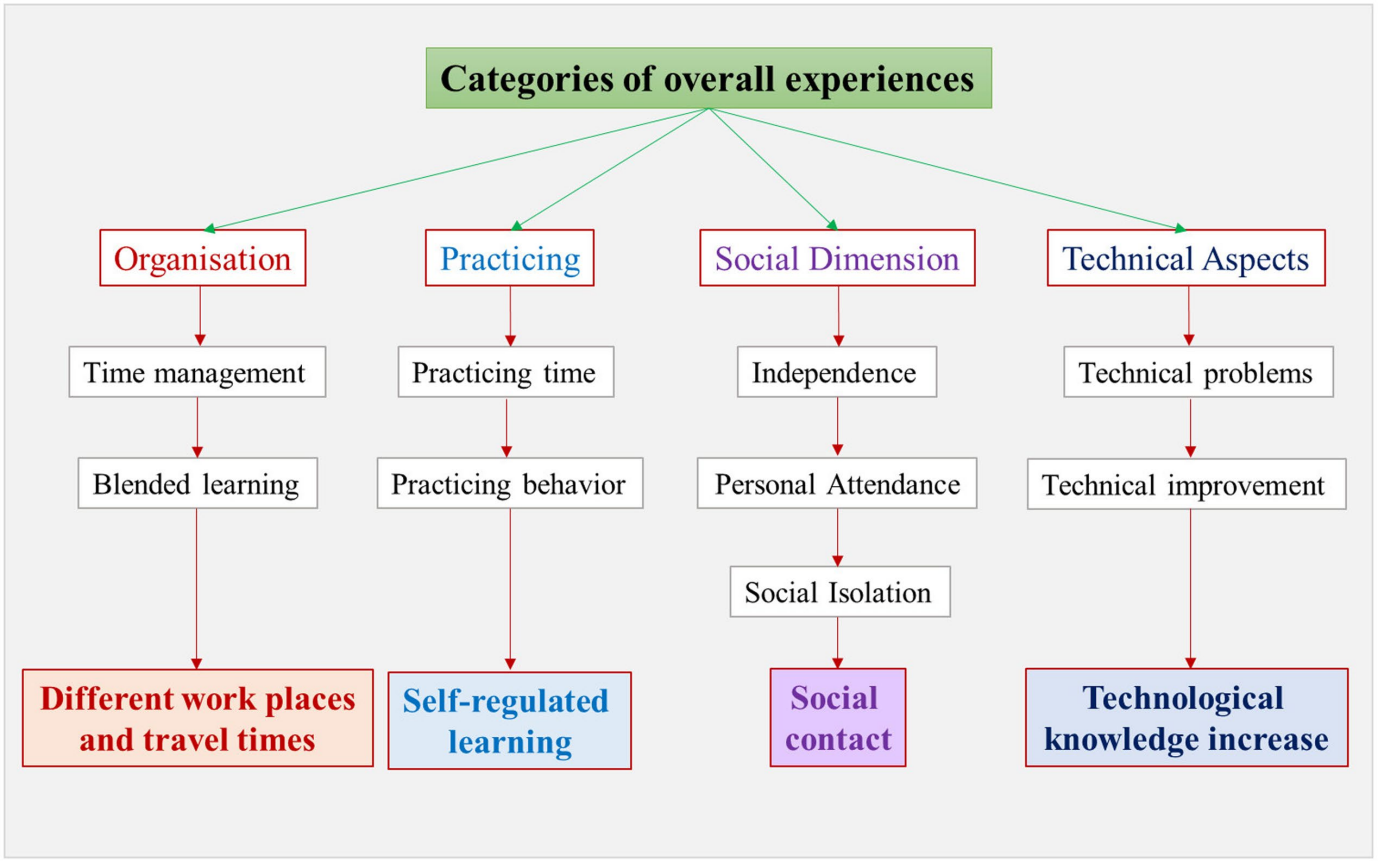
Inductive category system based on the open text responses of the participants.

The qualitative content analysis was used to analyze the students’ text-based statements in order to find out reasons for the students’ behavior during the three semesters examined. With the help of the summary content analysis, an inductive category system was designed, which forms the basis of the coding (see Figure 2), and led to the following interpretation.

## Results

### Overall experience of study

By use of the mixed-methods approach, we combined the quantitative and qualitative results. The quantitative data were looking at the individual overall experience of the different semesters, as well as practicing times, whereas the qualitative questions were considering self-regulated learning and self-organizational questions, practicing habits, as well as social and personal aspects. On this base, we developed four main categories: (1) “Organization,” (2) “Practicing,” (3) “Social Dimension,” and (4) “Technical Aspects” (Figure 2). Each of these categories was assigned codes that deepened the category and were based on the thematic aspects that the students were engaged in during the three semesters examined.

#### Overall experience

**Figure 3:**
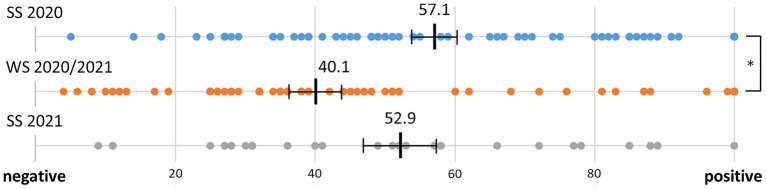
Ratings of the student’s experiences during each semester (the bold cross lines mark the mean value, the error bars present the standard error of the mean, ^*^p < 0.05).

The responses to the question of how the students experienced the semester compared to a non-coronavirus semester are shown in Figure 3. There was a significant main effect between the surveys [*F*(2,135) = 6.3, *p* = 0.002]. Individual *t*-tests against the neutral value of 50 yielded a significantly higher mean value in SS 2020 [*t*(62) = 2.26, *p* = 0.014], a significantly lower mean value in WS 20/21 [*t*(48) = −2.70, *p* = 0.005] and no significant difference in SS 2021 [*t*(25) < 1.0, n.s.].

In terms of the experience ratings of the semester compared to the previous semester, students in WS 20/21 rated that semester relative to the SS 2020 with an average of 75.2 (SD 18.1) and the SS 2021 relative to the WS 20/21 with an average of 74.1 (SD 20.7). Both ratings were significantly higher compared to the neutral value of 50 [WS 20/21: *t*(51) = 10.03, *p* < 0.001; SS 2021: *t*(25) = 5.93, *p* < 0.001].

#### Organization

Category 1, “Organization” arises from our qualitative data and focuses on two topics concerning the organization of student life during the three semesters.

During the lockdown semester of SS 2020, many students described a *self-oriented time management,* which turned out to be a typical feature of this semester. For example, one student commented that the semester had “more flexible scheduling and precise preparation of teachers,” referring to the concrete preparation of course contents. Another student wrote: “I found the freedom in my schedule relaxing.” Positive experiences with self-oriented time management were not mentioned for the hybrid-semester of WS 2020/21 or for the SS 2021, where all courses could once again be held with students in actual attendance.

A second main topic within this category was the experiences in the context of *blended learning,* focusing on room situations. During SS 2020 students indicated that they had experienced “local flexibility,” and the feeling of being “not spatially bound,” and that they liked “the flexibility in terms of space and time, so that I could arrange tasks myself and did not have to be constantly on the move between buildings.” The positive implications of solely online lessons were no longer reported by respondees for the hybrid semester of WS 2020/21. On the contrary a student wrote that its “organization was more complicated: [we had] new rules all the time.” Another student described how “[…] the online classes just do not work as well as face-to-face classes. Especially since I do not live so close to the university, switching between online and face-to-face teaching is very time-consuming.” Comparable experiences were not reported for the SS 2021.

The difficulties students experienced in spatial situations, represented in the term *different work places and travel times*, was reported by the students as a typical problem of blended learning, specifically the alternating between online and face-to-face classes.

#### Practicing

**Figure 4:**
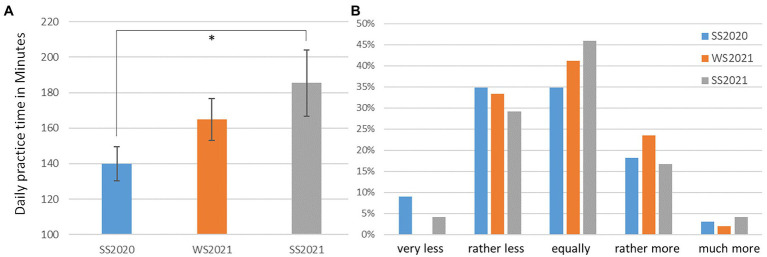
**(A)** Left: the mean daily practice time in each semester (error bars present the standard error of the mean, ^*^p < 0.05). **(B)** Right: distribution of the answers to the question of how the practice time differed from usual semesters before the pandemic.

The aspects *Practicing time* and *Practicing behavior* during the three different semesters can be derived from our quantitative and qualitative data (see Category 2 “Practicing” Figure 2). Across the three surveys, the main effect of the mean practice time was significant [Figure 4A, *F*(2,128) = 3.1, *p* = 0.049], showing 45 min more practice time per day in the SS 2021 than in the SS 2020, while the practice time in the WS 20/21 lies in between. The post-hoc analysis showed that the main effect across the three surveys reflected the difference between the SS 2020 and the SS 2021.

The responses to the question of how the practice time differs from usual semesters without coronavirus are also shown in Figure 4B. The average of the ratings was mainly around the answer of equal practice time. There was no significant main effect of the rating distribution between the surveys.

These results were related to those garnered from the personal statements of the students. In comparison to the other two semesters, especially in the SS 2020 students described a predominantly self-regulated practice behavior: One student wrote that during SS 2020: “I learned focused practicing,” while another student noted “a higher level of self-regulated working.” More than one student wrote about “concentrated self-study” during the lockdown period. “I practice in a more concentrated and constant and focused way.” And: “I worked in a concentrated and more organized way. Without pressure,” were further statements that described the practice behavior. Other students describe their practicing as follows: “I use body awareness as a tool for better practice. Goal-oriented practicing, with realistic and personal goals. This brings motivation for practice.” Or: “It is important to have good balance between practicing and breaks / other activities.” In conclusion, one student wrote: “The most important thing is to practice consciously. It does not depend on the amount of time to play better, but on the quality of the practice mainly.” Our qualitative data demonstrates an obvious predominance of *self-regulated learning* during the lockdown semester SS 2020.

#### Social dimension

The “Social Dimension” is an important category deriving from our qualitative data (Figure 2). The content analysis revealed three more aspects which were associated with this topic: *Independence, Personal Attendance* and *Social Isolation*.

Concerning the lockdown semester 2020 students wrote that they had a “high level of independent working,” as well as, “more free time,” and “free practice time,” or “more time to do sports,” leading to the code of “independence.”

*Personal attendance* for the SS 2020 was discussed in statements with remarks such as “good support from the teachers,” “online-courses were really good,” or: “I think that the university did a great job in coordinating online courses. They were solution-oriented and had patience when you had questions.”

However, at the end of SS 2020, social isolation was also indicated: “I cannot meet my teacher and colleagues,” or: “Contact with fellow students has been lacking.” Another one wrote “Above all, the lack of direct social contact in the courses or in the foyer of the university [was lacking].” Concerning the next semester WS 2020/21—where students of the University of Music Freiburg had instrumental lessons again and were allowed to use the university building for practicing—students commented: “You see people in the university again and can at least talk to them. One-to-one lessons can take place again, almost without restrictions.” Another socially-related topic during this period that was a source of regret was the fact that “no concerts have been taking place.” At the end of SS 2021, in which everything had been open again, students expressed great pleasure about “the possibility to play and go to concerts again,” and “that there are more options to meet face-to-face.” The statement of one student sums up this observation: “I enjoy my studies much more. It is more communicative and I no longer feel so pressured.” Another topic for the students was that they felt “more secure in life.” Negative points during this semester related mainly to daily life, such as: “wearing a mask all the time is exhausting,” or “it’s not all like before,” and they did not like “that not everyone follows the rules, e.g., ventilation breaks.”

Clearly, as shown by this qualitative data, *social contact* was an important topic for respondees.

#### Technical aspects

In the qualitative data, “Technical Aspects,” divided into *Technical Problems* and *Technical Improvement*, played an important role.

Technical problems were mainly addressed during SS 2020: “I had technical problems, internet problems, and not enough silence at home,” or as another student stated: “Online-courses always need a working internet connection.” Another student wrote that he had “technical problems, which hinder the learning process.” And: [online] “instrumental lessons […] were complicated.” During WS 2020/2021 this changed to *technical improvement*. Students wrote: “Technical implementation of teaching has improved” and concerning SS 2021: “Online possibilities became more diverse.” Overall, *Technological knowledge increase* emerged over the three semesters as an important topic.

## Discussion

In this study, music students were asked at the end of three different coronavirus-impacted semesters to report on their experiences during these semesters. The questions considered their general experiences, with additional focus on aspects of practicing. The mixed-methods approach of this study allowed the quantitative and qualitative data to be related to each other, showing that the qualitative statements of the students enabled a more differentiated interpretation of the quantitative results. This provides a profounder understanding and explanation of the findings.

### Comparison of students’ experiences under three different coronavirus conditions

A special feature of the present study is that three semesters that differ significantly in terms of their underlying conditions are compared with one another in terms of the personal experiences of the music students. However, it must be clearly stated that this is a comparison of three cross-sectional surveys and not a longitudinal study.

The overall experience of the music students in the three semesters compared to the pre-coronavirus semesters showed that the lockdown semester was rated significantly better than the following semester with blended learning and even better than the semester with full face-to-face teaching. This may seem surprising at first glance, but can be understood and discussed in a more differentiated way based on the qualitative results.

The answers from the students can be summarized in four categories, which crystallize to form important points in the three semesters: Organization, Practice, Social Contact and Technical Aspects.

It is interesting that there is not one theme that stands out at all three points in time. Rather, for each of the three semesters, a different topic is at the forefront for the students, which can only be explained by their personal statements in relation to the respective social and university-related underlying conditions prevailing in each semester concerned.

#### The lockdown semester – SS 2020

During this semester two topics were at the forefront of the students’ experiences: on the one hand they were challenged by social distancing, while on the other hand they had the chance to develop self-regulated learning techniques.

Even if the students suffered from social isolation during the lockdown semester and the music students in particular from the restrictions of one-to-one lessons, they also gained independence overall, saved time by eliminating the need to travel and experienced greater self-determination when practicing. The results of our study confirm the hypothesis of Habe et al. (2021) and Ozer and Ustun (2020) that music students gained constructive experiences during the lockdown. Therefore, we suspect that opportunities for music students also emerged, that can generate positive effects and create benefits, coming from the lockdown semester.

Schiavio et al. (2021) also found in their study of students at an Italian conservatory that distinct approaches to practicing and time management have emerged during the COVID-19 pandemic. Music students have developed more self-regulated practicing behaviors, resulting in a better understanding of their own learning and practicing (Antonini Philippe et al., 2020; Rosset et al., 2021).

Technical problems in the lockdown semester were also mentioned as disruptive in the present study. Students perceived the efforts in dealing with the new media and the commitment and attendance of the teachers as positive, especially in the lockdown semester, a finding that concurs with the research by Vladova et al. (2021).

#### The hybrid semester – WS 2020/21

The winter semester 2020/21 was characterized by the joy of the individual instrumental and vocal lessons taking place again, but also burdened by the still limited contact with other students. During this semester the question of how to deal with organizational requirements predominated due to the change between online and face-to-face classes. The students were also exposed to fundamental pressures, through strict societal restrictions (Stringency Index: Ritchie et al., 2020; Hale et al., 2021), which have led to additional challenges in the general population in Germany. Social isolation was still a topic for the students, but was not at the forefront of their minds anymore. As the students emphasize, switching between locations was complicated, and not always possible. It was complicated to coordinate online and face-to-face lessons. Even though students appreciated the opportunity to attend at least some courses in person, the hybrid format of the semester led to difficulties and confusion. Even more confusion was experienced by the constantly changing social and organizational conditions imposed from outside to which the University of Music Freiburg had to react in a regular manner.

Looking at all the students’ comments, they report less self-determination. However, they state an improvement in the technology of online offerings. In agreement with Li et al. (2021), Rucsanda et al. (2021), as well as Güsewell and Terrien (2021), a variety of learning formats in blended learning were rated positively, provided that the organizational requirements were adequately handled. This appears to be an important finding for future teaching practice.

#### The mostly opened semester – SS 2021

During this semester students were focused on two topics: the resurgence of social contacts, as well as a better handling with digital communication formats. The 2021 summer semester was mainly characterized by the positive experience of social contacts in the large ensembles and orchestras that were taking place again, as well as meeting fellow students on a regular basis once again. This points to the importance of social contact as an integral part of university life and activity. Added to this was the increase in technological knowledge on the part of students. Practicing approached the pre-coronavirus levels again.

### Practice time and behavior

As some studies have already discussed, *practice time* decreased during the lockdown semester. However, Nusseck and Spahn (2021) found that while music students spent less time practicing, some aspects of self-regulated learning increased. The students’ statements confirm this observation, noting that “I developed more routines,” and “I gained more awareness of my body and was able to practice in a more autonomous way.” Another student told us that he found out “that I have to take breaks.” Practicing during lockdown was also described as “more focused and concentrated.”

The quantitative data show that the majority of the students rate their own practice time—always in relation to the previous semester—as constant. However, if one relates this answer to the stated practice time in hours, it becomes apparent that they did not spend the same amount of time practicing, but rather an increasing number of minutes per day.

While the average number of hours spent practicing in the summer semester 2020 was lower than in the previous semester, the number of hours spent practicing increased somewhat in the winter semester 2020/21 and continued to increase until the summer semester 2021. If the students’ quantitative statements are also taken into account, it can be concluded that although they spent significantly less time practicing during the lockdown semester, they did not notice this and expressed positive opinions about practicing. They were satisfied with the free time they had gained, were able to practice in a self-determined and self-regulated manner, yet also organized all their time themselves. The result was that the students achieved a degree of satisfaction with regard to their self-regulated time management, which they obviously did not have or did not know before in this form.

The self-regulated learning approach (Boekaerts, 1999; Hatfield et al., 2016; McPherson et al., 2017; Schunk and Greene, 2017) is characterized by students determining and monitoring the learning process themselves. The model is primarily based on motivation and the desire to learn. Self-regulated learning contains three different strategies: metacognitive, cognitive, and resource management-related (Boekaerts, 1999; Kopp and Mandl, 2011). Cognitive strategies are concerned with organizing, elaborating, reviewing and repeating what was learned. Metacognitive strategies are designed to guide, control, and regulate the use of appropriate learning strategies. Resource management strategies refer to aspects that can be directed both by the students themselves and by others. In the case of our study, the music students’ resource management changed due to the coronavirus, which in turn caused them to reallocate and adapt their own resources to the new situation. According to Hatfield et al. (2016), and based on the students’ personal statements, we found that students changed their practice behaviors during the lockdown, adapting them through new goal setting, as well as enhanced self-efficacy.

### Practical implications for music education

The results of the cross-sectional study in three semesters with different framework conditions focus on different aspects that can play an important role in music education.

From our point of view, an important result is the confirmation that self-regulated learning can be promoted if music students have more time for self-organization. This complements the results of other studies with the aspect that self-determination can arise through less control and less goal-oriented specifications, without direct instruction for self-regulated learning. At the same time, our results make it clear that external structures can have a decisive influence on practice behavior. In the practical implementation in music education, the frequency of the individual lesson must be reflected precisely against this background. This is often stipulated in the assignment plan, regardless of the individual learning curve of the student. In a more flexible teaching setting, the frequency and duration of teaching could also be more individually regulated and adjusted.

With regard to the combination of courses in online and face-to-face formats, the important result was that new organizational aspects must be taken into account. Since there are not enough computer workstations for students at the University of Music, online formats are usually used by students at home. The transport time, which is necessary when changing between online lessons at the place of residence and face-to-face lessons at the University of Music, must be scheduled in the study plan.

Our results also give clear indications that social contacts during studies, both with teachers and with peers, are a central resource for the students’ psychological well-being and their performance. While this finding is by no means new and confirms the role of social support in health, it may raise the question of whether face-to-face arts classes can be replaced by online formats. Our results provide arguments for the fact that face-to-face encounters between students cannot be replaced by online formats.

### Limitations of the study

The study is based on a small sample, which, while representative for the University of Music Freiburg, has limitations in transferring to other universities.

Our questionnaire took place at three points in time that were very different, meaning that we were unable to collect the same number of answers for all three times. In particular, our third survey was answered by only a few students.

The design is cross-sectional, not longitudinal. This also has to be taken into account.

The experience rating scales have some methodological issues. The labeling only of the extremes (negative/positive) might have caused some misunderstanding. It would have been better to also label the neutral value in the middle with “equally experienced” to clarify the scale. Since the scale is a relative measure, it was not clear how the students would have responded without pandemic context to provide comparable ratings. It was assumed that the rating would be in the middle for similar semesters as it has been found for the SS 2021.

## Conclusion

The semesters impacted by the coronavirus have given students new experiences that can be used in the future. Music students state that they have benefited from online formats outside of the one-to-one lessons. The technological knowledge on the part of students and teachers and their technical equipment have increased and improved, respectively, during the coronavirus semesters. When offering blended learning formats, university educators must take into account organizational aspects such as spatial distances, locations etc. As expected, the great importance of social contact between teachers and peers, and among peers, has been confirmed. At the same time, students show more self-regulatory behavior when they are less controlled by face-to-face formats. Both must be reconciled in the future. The results of the present study show that the experiences of the students from the semesters during the coronavirus pandemic provide constructive starting points for future teaching.

## Data availability statement

The raw data supporting the conclusions of this article will be available from the authors, without undue reservation.

## Ethics statement

Ethical review and approval was not required for the study on human participants in accordance with the local legislation and institutional requirements. Written informed consent for participation was not required for this study in accordance with the national legislation and the institutional requirements. The authors declare that the data collection was conducted within the framework of the “Netzwerk Musikhochschulen,” which conducts regular evaluations according to the standards of quality management.

## Author contributions

All authors listed have made a substantial, direct, and intellectual contribution to the work and approved it for publication.

## Funding

We acknowledge support by the Open Access Publication Fund of the University of Freiburg.

## Conflict of interest

The authors declare that the research was conducted in the absence of any commercial or financial relationship that could be construed as a potential conflict of interest.

## Publisher’s note

All claims expressed in this article are solely those of the authors and do not necessarily represent those of their affiliated organizations, or those of the publisher, the editors and the reviewers. Any product that may be evaluated in this article, or claim that may be made by its manufacturer, is not guaranteed or endorsed by the publisher.
